# Effect of High Myopia and Cataract Surgery on the Correlation Between Diabetic Retinopathy and Chronic Kidney Disease

**DOI:** 10.3389/fmed.2022.788573

**Published:** 2022-06-01

**Authors:** Huiqian Kong, Siwen Zang, Yijun Hu, Zhanjie Lin, Baoyi Liu, Xiaomin Zeng, Yu Xiao, Zijing Du, Wu Guanrong, Yun Ren, Ying Fang, Yang Xiaohong, Honghua Yu

**Affiliations:** ^1^Department of Ophthalmology, Guangdong Provincial People's Hospital, Guangdong Eye Institute, Southern Medical University, Guangdong Academy of Medical Sciences/The Second School of Clinical Medicine, Guangzhou, China; ^2^Refractive Surgery Center, Guangzhou Aier Eye Hospital, Aier Institute of Refractive Surgery, Guangzhou, China; ^3^Aier School of Ophthalmology, Central South University, Changsha, China; ^4^Graduate School, Shantou University Medical College, Shantou, China

**Keywords:** diabetic retinopathy, chronic kidney disease, high myopia, cataract surgery, fundus image

## Abstract

**Purpose:**

To investigate the effect of high myopia and cataract surgery on the grading of diabetic retinopathy (DR) and their roles in the correlation between DR and chronic kidney disease (CKD).

**Methods:**

A total of 1,063 eyes of 1,063 diabetic patients were enrolled. We conducted binary and multiple multivariate regressions to analyze the ocular and systemic risk factors of DR. Based on the presence of myopia and history of cataract surgery, we divided the cases into four subgroups, namely those with high myopia, with the history of cataract surgery, with both conditions, and with neither, then determined the correlation between the stages of DR and CKD in each subgroup.

**Results:**

In the binary analysis, high myopia was identified as the protective factor for DR odds ratio (OR): 0.312 [95% confidence interval (CI): 0.195–0.500, *p* < 0.001], whereas cataract surgery was one of the independent risk factors for DR [OR: 2.818 (95% CI: 1.507–5.273), *p* = 0.001]. With increased stages of DR, high myopia played an increasingly protective role [mild non-proliferative DR (NPDR), OR = 0.461, *p* = 0.004; moderate NPDR OR = 0.217, *p* = 0.003; severe NPDR, OR = 0.221, *p* = 0.008; proliferative DR (PDR), OR = 0.125, *p* = 0.001], whereas cataract surgery became a stronger risk factor, especially in PDR (mild NPDR, OR = 1.595, *p* = 0.259; moderate NPDR, OR = 3.955, *p* = 0.005; severe NPDR, OR = 6.836, *p* < 0.001; PDR, OR = 9.756, *p* < 0.001). The correlation between the stages of DR and CKD in the group with neither high myopia nor cataract surgery history was the highest among all subgroups.

**Conclusion:**

High myopia was a protective factor, whereas cataract surgery is a risk factor for DR, and both factors showed stronger effects throughout the (natural disease) grading of DR. The stages of DR and CKD showed a higher correlation after adjustment of the ocular confounding factors.

## Introduction

Diabetic retinopathy (DR) is one of the most frequent microvascular complications of diabetes, affecting approximately 25% of patients with type 2 diabetes mellitus, and it has become a leading cause of visual impairment and blindness (1–4). The annual incidence of DR ranges from 2.2 to 12.7%, and the rate of DR grading is 3.4–12.3% (2). The prevalence of DR and sight-threatening diabetic retinopathy (STDR) in diabetes was 27.9 and 12.6%, respectively in this multi-hospital-based population across China (5). With the rising incidence and prevalence of DR, ascending costs of DR screening program among diabetic patients are posing higher burden to the public health system (6).

In the past several years, reports have revealed that the kidney and eye have a strong connection, since they share common risk factors, similar biological mechanisms and pathways (7). Our previous study revealed that chronic kidney disease (CKD) was positively related with DR (*r* = 0.264, *p* < 0.001) (8), which was consistent with the findings of other studies (9–11). However, other studies hold the views that the correlation between DR and CKD remains uncertain (12, 13) and consider that low glomerular filtration rate (GFR), a commonly used index for CKD evaluation, is not significantly associated with DR (12). Therefore, we aimed to find out the factors that may affect the correlation between DR and CKD. DR and CKD share many common systemic risk factors, such as long diabetes duration, high hemoglobin A1C (14), high pulse pressure (15), high myopia (16), intraocular surgery (17), and so forth. However, the severity of DR may be affected by ocular factors (such as high myopia and cataract surgery) that are not associated with CKD grading (7, 18, 19).

Thus, we considered that ocular factors may be the confounding factors that contribute to the non-synchronicity between the stages of DR and CKD. In this study, we investigated the ocular risk factors of DR and identified their effects on the correlation between DR and CKD.

## Methods

### Study Population

This research is a retrospective cross-sectional study in which the information of 1,866 diabetic patients who had received ophthalmic consultation at Guangdong Provincial People's Hospital from September 2016 to February 2020 were collected. Patients in accordance with the following conditions were excluded: under 18 years old; loss of information; complicated with serious systemic disease, including immunodeficiency disease, malignant disease, and metabolic diseases other than diabetes; presence of ocular disease possibly affecting ocular circulation, such as glaucoma, uveitis, and fundus diseases other than DR; history of receiving DR treatments including anti-vascular endothelial growth factor (VEGF) injection, retinal laser therapy, and operation. Our study included patients with hypertension, but excluded patients with hypertension complications, such as hypertensive heart disease, hypertensive nephropathy, and hypertensive retinopathy. In accordance with the abovementioned criteria, 1,063 diabetic patients were enrolled. Our study was conducted in accordance with the Declaration of Helsinki and was approved by the Ethics Committee of Guangdong Provincial People's Hospital (NO. GDREC2020069H).

### Data Collection

The information of patients was collected from electronic medical records and corresponding image storage. Systemic indicators were collected, which included gender, age, duration of diabetes, total cholesterol, apolipoprotein B, apolipoprotein AI, low-density lipoprotein, triglyceride, glycosylated hemoglobin, urinary albumin creatinine ratio (UACR), estimated glomerular filtration rate (eGFR), history of hypertension, urea, magnesium, sodium, potassium (K), serum creatinine (blood Cr), chlorine, lactate dehydrogenase (LDH), total protein, conjugated bilirubin, and cholinesterase level.

The ophthalmic indicators acquired from ophthalmic consultation included the best-corrected visual acuity, intraocular pressure, dilated fundus examination, and fundus photography (non-stereoscopic 45°photograph of the central fundus and of the optic disc), using A non-mydriatic retinal camera (Topcon TRC; Topcon, Tokyo, Japan) (20). High myopia was defined as a spherical equivalent of <−6.00D (21). The history of cataract surgery was defined having cataract surgery on the enrolled eye at least a months before.

### Assessment of DR

Based on medical records and fundus images, we performed DR grading. DR grading was performed by two ophthalmologists (H.K., Y.F.); any inconsistency would be judged by a retinal specialist (Y.H.). The analysis was based on patients. Only one eye was enrolled in each patient, and the more severe was adopted if the severities of the two eyes were different. In accordance with the International Clinical Diabetic Retinopathy and Diabetic Macular Oedema Disease Severity Scales (22), DRs were divided into five stages: normal fundus, mild non-proliferative DR (NPDR), moderate NPDR, severe NPDR, and proliferative DR (PDR).

### Definition and Grading of CKD

eGFR and UACR are the major indicators commonly used to evaluate renal function. Therefore, in accordance with the Chronic Kidney Disease Epidemiology Collaboration creatinine equation based on eGFR, CKD was classified into 1–5 stages (23): stage 1, eGFR ≥90 mL/min/1.73 m^2^; stage 2, eGFR: 60–89 mL/min/1.73 m^2^; stage 3, eGFR: 30–59 mL/min/1.73 m^2^; stage 4, eGFR: 15–29 mL/min/1.73 m^2^; stage 5, eGFR<15 mL/min/1.73 m^2^; severe nephropathy stage, eGFR<60 mL/min/1.73 m^2^. Using the United States National Kidney Foundation based on UACR, CKD was also divided into the following five stages: stage 1, UACR<10 mg/g; stage 2, UACR: 10–29 mg/g; stage 3, UACR: 30–299 mg/g; stage 4, UACR: 300–999 mg/g; stage 5 UACR ≥1,000 mg/g. UACR stages 3–5 is defined as severe nephropathy stages (24).

### Statistical Analysis

We performed all analyses using SPSS version 23.0 (SPSS, Chicago, Illinois, USA). Continuous data were characterized as mean ± standard deviation or median (interquartile ranges), whereas categorical variables were denoted as numbers and percentages (%). Chi-square test, *t*-test, and Mann-Whitney U test were used to test the differences between patients with DR and those without DR. Univariate and multivariate binary logistic regressions were used to determine the systemic and ocular risk factors of DR. Ocular factors selected from the last step were analyze by multiple logistic regression to test their influence on different stages of DR. Moreover, the correlation between the stages of CKD and DR was performed using Spearman's correlation analysis.

## Results

### Baseline Characteristics

Of the 1,063 patients, the mean age was 59.3 ± 14.5 years old, and 559 (54.4%) of them were male. A total of 430 (40.5%) patients had DR in at least one eye, while 633 (59.5%) had no DR in either eye.

Comparisons of baseline characteristics between with- and without-DR groups were shown in Table 1. Compared with the without-DR group, the patients with DR were more likely to be female (*p* = 0.038), without high myopia (*p* = 0.004), have cataract surgery history (*p* < 0.001), longer durations of diabetes (*p* < 0.001), with a history of hypertension (*p* < 0.001), lower total protein (*p* = 0.036), lower conjugated bilirubin (*p* < 0.001), lower K (*p* = 0.002), and higher blood Cr (*p* < 0.001), higher LDH (*p* < 0.001), and higher urea (*p* < 0.001).

**Table 1:** Characteristics and differences between DR and without DR patients.

	**DR**	**Without DR**	***p*-value**
Patients, *n* (%)	430 (40.5)	633 (59.5)	
Age (years)	64.0 (55.0–71.0)	57.0 (48.0–68.3)	<0.001^*^
Sex, male, *n* (%)	214 (49.8)	359 (56.7)	0.038^*^
High myopia, *n* (%)	47 (10.9)	117 (18.5)	0.004^*^
Cataract surgery, *n* (%)	58 (13.5)	17 (2.7)	<0.001^*^
Duration of diabetes, *n* (%)				
<5 years	130 (30.2)	309 (48.8)	<0.001^*^
5–10 years	122 (28.4)	158 (25.0)	<0.001^*^
10–20 years	145 (33.7)	135 (21.3)	<0.001^*^
>20 years	24 (5.6)	30 (4.7)	<0.001^*^
Glycosylated hemoglobi*n* (%)	9.6 (8.0–11.2)	9.2 (7.5–11.2)	0.161
Stage of CKD, <60 mL/min/1.73 m^2^, *n* (%)	156 (36.3)	79 (12.5)	<0.001^*^
Stage of UACR, *n* (%)				
Stage 1	117 (27.2)	397 (62.7)	<0.001^*^
Stage 2	69 (16.0)	129 (20.4)	<0.001^*^
Stage 3	121 (28.1)	85 (13.4)	<0.001^*^
Stage 4	46 (10.7)	16 (2.5)	<0.001^*^
Stage 5	77 (17.9)	6 (0.9)	<0.001^*^
Hypertension, with, *n* (%)	163 (37.9)	274 (43.3)	<0.001^*^
Blood Cr (μmol/L)	87.7 (68.9–124.0)	73.0 (59.8–87.2)	<0.001^*^
Total protein (g/L)	64.5 ± 6.7	65.5 ± 6.1	0.036^*^
LDH (U/L)	178.0 (154.0–206.0)	162.0 (141.0–190.0)	<0.001^*^
Conjugated bilirubin (μmol/L)	2.1 (1.5–3.3)	2.5 (1.8–3.4)	<0.001^*^
Cholinesterase (U/L)	8,051.0 (6,626.0–9,372.0)	8,167.5 (6,833.0–9,654.8)	0.072
LDL (mmol/L)	3.0 (2.6–3.7)	3.1 (2.5–3.7)	0.614
HDL (mmol/L)	1.0 (0.8–1.2)	1.0 (0.9–1.2)	0.622
Triglyceride (mmol/L)	1.5 (1.1–2.2)	1.5 (1.0–2.3)	0.675
Total cholesterol (mmol/L)	4.8 (3.9–5.6)	4.8 (4.0–5.6)	0.633
Apolipoprotein B100 (g/L)	1.0 (0.8–1.1)	0.9 (0.8–1.1)	0.586
Apolipoprotein AI (g/L)	1.1 (1.0–1.3)	1.2 (1.0–1.3)	0.422
Urea (mmol/L)	6.1 (4.7–8.4)	5.1 (4.1–6.4)	<0.001^*^
Na (mmol/L)	138.4 (135.9–140.4)	138.7 (135.8–140.6)	0.808
K (mmol/L)	3.8 (3.6–4.2)	3.8 (3.5–4.1)	0.002^*^
Mg (mmol/L)	0.8 (0.8–1.0)	0.8 (0.8–1.0)	0.533
Cl (mmol/L)	103.4 (101.0–106.2)	104.1 (101.4–106.1)	0.519

*Results are expressed as mean±SD, percentages or as medians (IQR)*.

*DR, Diabetic retinopathy; CKD, Chronic kidney disease; UACR, Urinary protein creatinine ratio; LDL, Low density lipoprotein; HDL, High density lipoprotein*.

*p-values were obtained by Mann-whitney test, Chi-square test, and binary univariate regression*.

* *Statistically significant difference at p < 0.05*.

### Systemic and Ocular Risk Factors of DR

Results of the multivariate binary logistic regression analysis were shown in Table 2. The systemic risk factors for DR were older age (odds ratio (OR): 1.026, 95% confidence interval (CI): 1.012–1.042; *p* < 0.001), longer duration of diabetes (OR: 1.164, 95% CI: 1.090–1.708; *p* = 0.007), with a history of hypertension (OR: 2.189, 95% CI: 1.504–3.175; *p* = 0.008), higher serum creatinine value (OR: 1.009, 95% CI; 1.005–1.013; *p* < 0.001), and ocular risk factor was cataract surgery (OR: 2.106, 95% CI: 1.507–5.273; *p* = 0.001), whereas the protective factor for DR was high myopia (OR: 0.274, 95% CI: 0.170–0.442; *p* < 0.001).

**Table 2:** Multivariate Binary logistic regression analysis for risk factors of DR.

**Factors**	**OR (95%CI)**	***p*-value**
Age (per year)	1.026 (1.012–1.042)	<0.001^*^
Sex (male vs. female)	1.323 (0.939–1.863)	0.110
High myopia (with vs. without)	0.274 (0.170–0.442)	<0.001^*^
Cataract surgery (with vs. without)	2.106 (1.507–5.273)	0.001^*^
Duration of diabetes (per 5 years)	1.164 (1.090–1.708)	0.007^*^
Hypertension (with vs. without)	2.189 (1.504–3.175)	0.008^*^
Total protein (per g/L)	1.001 (0.976–1.027)	0.922
Conjugated bilirubin (perμmol/L)	0.914 (0.814–1.027)	0.131
Blood Cr (perμmol/L)	1.009 (1.005–1.013)	<0.001^*^
LDH (per U/L)	1.003 (1.000–1.006)	0.064
K (per mmol/L)	0.992 (0.977–1.008)	0.331

*Blood Cr, Blood Serum creatinine; LDH, Low density lipoprotein; K, Blood potassium*.

* *Statistically significant difference at p < 0.05*.

### Effect of High Myopia and Cataract Surgery on Different Stages of DR

**Figure 1:**
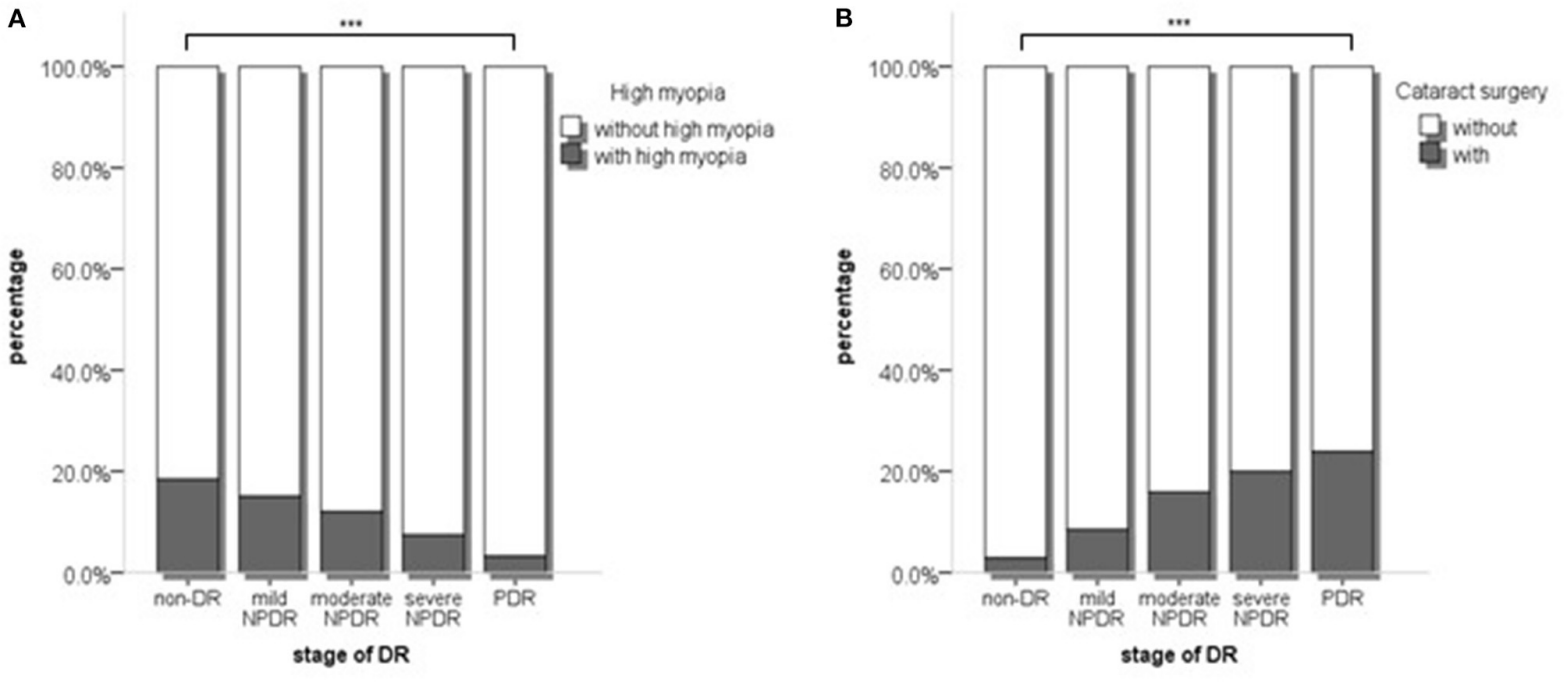
**(A)** The proportion of high myopia and non-high myopia in different stages of DR; **(B)** The proportion of with and without cataract surgery in different stages of DR. *p* values were acquired from Chi-square test. DR, Diabetic retinopathy. ***p < 0.001.

Firstly, we compared the proportion of patients with high myopia and cataract surgery in different DR stages. The proportions of high myopia declined with 18.4, 15.0, 12.0, 7.4, and 3.2% (*p* < 0.001, Figure 1A), whereas the proportion of cataract surgery increased by 2.9, 8.5, 15.9, 20.0, and 23.9%, in non-DR, mild NPDR, moderate NPDR, severe NPDR, and PDR group, respectively (*p* < 0.001, Figure 1B) (*p* < 0.001). The influence of high myopia and cataract surgery on different stages of DR was shown in Table 3. With the grading of DR, high myopia played an increasingly protective role (mild NPDR, OR = 0.461, *p* = 0.003; moderate NPDR, OR = 0.217, *p* = 0.003; severe NPDR, OR = 0.221, *p* = 0.008; PDR, OR = 0.125, *p* = 0.001). On the contrary, cataract surgery became a higher risk factor in PDR than in any stage of NPDR (mild NPDR, OR = 1.595, *p* = 0.258; moderate NPDR, OR = 3.955, *p* = 0.005; severe NPDR, OR = 6.836, *p* < 0.001; PDR, OR = 9.756, *p* < 0.001).

**Table 3:** Analysis of risk factors in different stages of DR.

**Factors**	**OR (95%CI)**	***p*-value**
**Mild NPDR**
High myopia	0.461 (0.274–0.777)	0.003^*^
Cataract surgery	1.595 (0.709–3.589)	0.258
**Moderate NPDR**
High myopia	0.217 (0.080–0.590)	0.003^*^
Cataract surgery	3.955 (1.523–10.265)	0.005^*^
**Severe NPDR**
High myopia	0.221 (0.072–0.688)	0.008^*^
Cataract surgery	6.836 (2.672–17.490)	<0.001^*^
**PDR**
High myopia	0.125 (0.035–0.446)	0.001^*^
Cataract surgery	9.756 (4.216–22.574)	<0.001^*^

*Adjusted by age, sex, diabetic duration, hypertension, blood serum creatinine, total protein, conjugated bilirubin, low density lipoprotein, and total cholesterol*.

*NPDR, Non proliferative diabetic retinopathy; PDR, Proliferative diabetic retinopathy*.

* *Statistically significant difference at p < 0.05*.

### Effect of High Myopia and Cataract Surgery on the Correlation Between the Stages of DR and CKD

**Figure 2:**
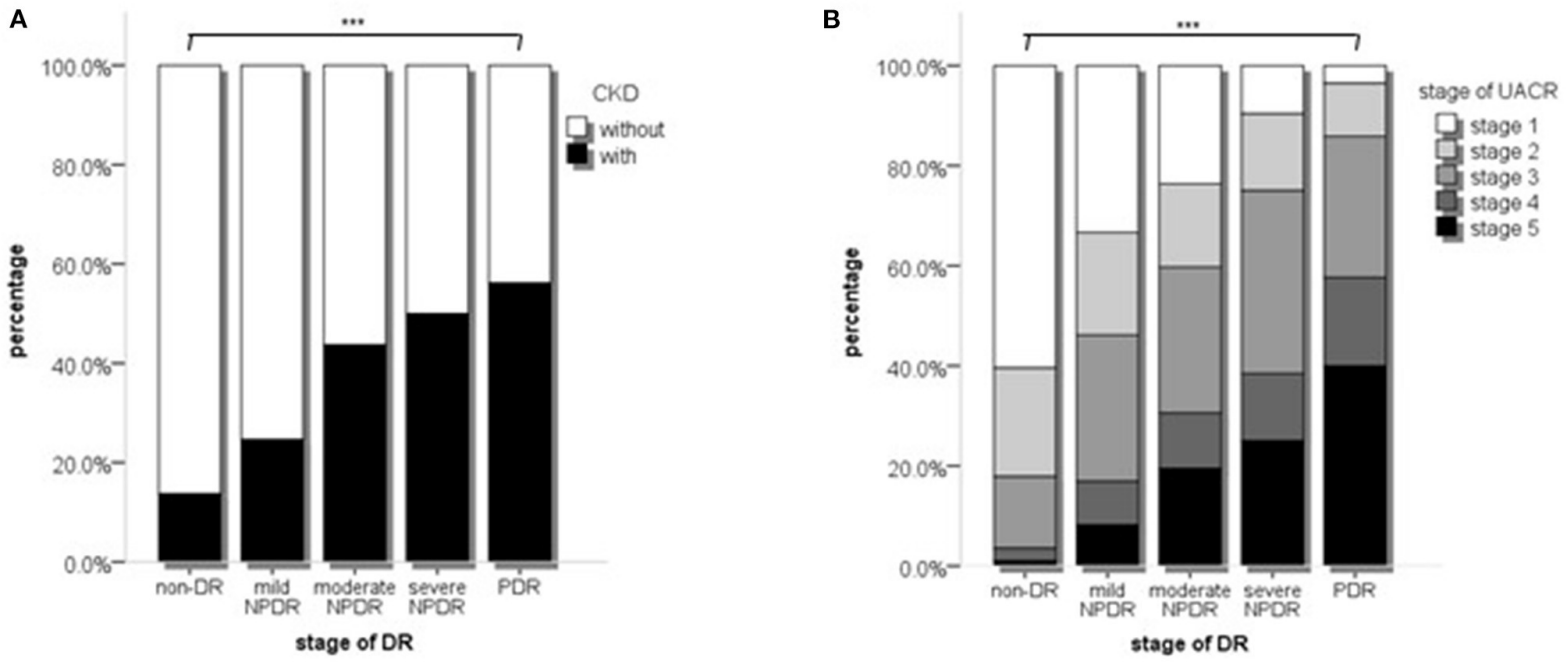
The proportions of different stages of CKD or UACR in different stages of DR. **(A)** The proportions of different stages of CKD in different stages of DR; **(B)** The proportion of different stages of UACR in different stages of DR stages. *p* values were acquired from Chi-square test. CKD, Chronic kidney disease; UACR, Urinary protein; DR, Diabetic retinopathy. ***p < 0.001.

Thirdly, we compared the proportions of severe nephropathy in different DR stages. The patients with eGFR<60 mL/min/1.73 m^2^ accounted for 13.7, 24.6, 43.7, 50.0, and 56.2% of the non-DR, mild NPDR, moderate NPDR, severe NPDR, and PDR groups, respectively (*p* < 0.001, Figure 2A). The patients with UACR stages 3–5 accounted for 17.9, 46.2, 59.7, 75.0, and 85.6% (*p* < 0.001, Figure 2B).

We suspected that the ocular independent risk and protective factors may lead to non-synchronous grading between DR and CKD. Hence, on the basis of the risk and protective factors, we performed subgroup analysis to prove our hypothesis. We divided the study population into four subgroups, including those with high myopia, with cataract surgery, with both conditions, and with neither. Correlation analysis for the stages of DR and CKD was performed among all the subgroups. The group with neither condition showed the strongest correlation between the stages of DR and CKD (Table 4) (stages of DR and eGFR, *r* = 0.366, *p* < 0.001; stage of DR and UACR, *r* = 0.521, *p* < 0.001), and the results of partial relation was consistent with this finding (stages of DR and eGFR, *r* = 0.374, *p* < 0.001; stage of DR and UACR, *r* = 0.531, *p* < 0.001).

**Table 4:** The correlation between the stages of DR and CKD in the four subgroups.

	**Stages of DR and eGFR**		**Stages of DR and UACR**	
	***r***	***p*-value**	***r***	***p*-value**
Subgroup-analysis				
With high myopia	0.338	<0.001^*^	0.304	<0.001^*^
With cataract surgery	0.214	0.048^*^	0.415	0.010^*^
With both	0.263	<0.001^*^	0.019	<0.001^*^
With neither	0.366	<0.001^*^	0.521	<0.001^*^
Partial relation				
Adjust both^#^	0.374	<0.001^*^	0.531	<0.001^*^

*CKD, Chronic kidney disease; UACR, Urinary protein; DR, Diabetic retinopathy*.

# *Adjusted by high myopia and cataract surgery*.

* *Statistically significant difference at p < 0.05*.

## Discussion

In this study, we discovered that high myopia was a protective factor in the grading of DR [OR = 0.274 (0.170–0.442), *p* < 0.001]. Although a number of studies revealed that high myopia was negatively related with DR (16, 25–28), our work indicated that the influence of high myopia became more significant during the grading of DR. The mechanism may involve two aspects. Firstly, thin retinal venular diameter and decreased retinal function, which consumes relatively less oxygen and becomes hypoxic in the presence of diabetes in high myopia, play a significant role in the protective effect of high myopia against DR (25, 26). Lin et al. reported that eyes with high myopia had significantly less severity of microaneurysms, less spot hemorrhages, and less presence of hard exudates and intraretinal microvascular abnormality (25). Akiba et al. also proved that DR showed less incidence in patients with posterior vitreous detachment, which always occurs in high myopia cohorts (29). On the other hand, eyes with myopia have lower concentration levels of VEGF, than those without high myopia, thus accelerating the grading of DR. Based on this, the grading of DR might be slower in patients with high myopia, but even so, we still found PDR in patients with high myopia. Therefore, regular fundus examinations are still needed. Secondly, based on the binary logistic regression and multiple regression analyses, the patient after cataract surgery is likely to develop DR (OR = 2.818, 95% CI: 1.507–5.273; *p* = 0.001). Several reports revealed that cataract surgery might accentuate intraocular inflammation, especially in the diabetic population, because of the high levels of systemic inflammation in these patients (17, 30, 31). Dong et al. also indicated changes in aqueous humor factors, that is, the levels of interleukin (IL)-1β, IL-6, IL-8, interferon-inducible protein 10, monocyte chemoattractant protein*-*1, and VEGF increased in patients with diabetes after cataract surgery (32). In addition, patients with severe DR are more likely to be in poor blood glucose control, which will also aggravate the formation of cataract (33). Therefore, more attention on the fundus condition should be paid to diabetes patients when planning cataract surgery. Choosing the appropriate timing of cataract surgery and more frequent fundus examination may be useful to prevent the occurrence and grading of diabetic retinopathy.

Previously, the relationship between the DR and CKD was controversial (7, 14). In diabetic populations, Sabanayagam et al. revealed that DR was associated with CKD only in patients with proteinuria (13). In another aspect, numerous studies showed that the grading of CKD is a risk factor for DR (4, 6, 14). However, a limited number of researches had considered the confounders of DR, such as high myopia and cataract surgery. Thus, we performed subgroup analysis and partial relation to adjust the confounders, and the results revealed strong correlations between CKD and DR and between UACR and DR. Our study also revealed that the influence of high myopia and cataract surgery varied in different stages of DR. Thus, these two factors can affect the accuracy of the prediction of CKD from fundus photographs in diabetic patients.

With the innovation of artificial intelligence technology, in recent years, there are many studies have applied artificial intelligence to the diagnosis, treatment and prediction of fundus disease (34, 35), including detection of CKD from retinal photographs by deep learning algorithm (36). Based on our findings, ocular factors such as high myopia and cataract surgery should be implemented into the deep learning models to improve the performance. Therefore, our work could serve as a reminder to some extent, and adding gaps to the comprehensive management of diabetic patients.

Our studies had several limitations. Firstly, this research is a cross-sectional study, thus further evidence is needed from prospective cohort studies. Secondly, we considered only two ocular confounders, and we will continue deepening the study to investigate other existing ocular factors.

In conclusion, high myopia and cataract surgery have different effects on different stage of DR. These two factors can influence the correlation between DR and kidney disease, thus affecting the accuracy of CKD detection from fundus photos.

## Data Availability Statement

The raw data supporting the conclusions of this article will be made available by the authors, without undue reservation.

## Ethics Statement

Written informed consent was obtained from the individual(s) for the publication of any potentially identifiable images or data included in this article.

## Author Contributions

HK, SZ, and YH: project execution and article writing. YXiaoh and HY: project design. ZL, BL, XZ, YXiao, ZD, WG, YR, and YF: data collection. All authors contributed to the article and approved the submitted version.

## Funding

This study was supported by the National Natural Science Foundation of China (81870663 to HY), the Science and Technology Program of Guangzhou (202002030074 to HY and 202002020049 to YXiaoh), the Outstanding Young Talent Trainee Program of Guangdong Provincial People's Hospital (KJ012019087 to HY), the talent introduction fund of Guangdong Provincial People's Hospital (Y012018145 to HY), the Technology Innovation Guidance Program of Hunan Province (2018SK50106 to YH), and the Science Research Foundation of Aier Eye Hospital Group (AR1909D2 and AM1909D2 to YH). The sponsors or funding organizations had no role in the design or conduct of this research.

## Conflict of Interest

The authors declare that the research was conducted in the absence of any commercial or financial relationships that could be construed as a potential conflict of interest.

## Publisher's Note

All claims expressed in this article are solely those of the authors and do not necessarily represent those of their affiliated organizations, or those of the publisher, the editors and the reviewers. Any product that may be evaluated in this article, or claim that may be made by its manufacturer, is not guaranteed or endorsed by the publisher.
